# Literary Notes

**Published:** 1907-06

**Authors:** 


### LITERARY NOTES

THE *Index Medicus* is sending out an appeal to the medical profession for support. We quote the following paragraph from its circular:

The subscription price of the last five volumes of the First Series of the *Index Medicus* was $25 a year. In setting the annual price of the present series at $5, though it bears no proportion to the cost of production, the Carnegie Institution of Washington anticipated that physicians, health officers, librarians and statisticians would very generally subscribe for the journal. This expectation has not been realised. Unless it appears that the *Index Medicus* is of greater service to the medical profession and can help to support itself to a greater extent than in the past, it may become advisable to discontinue its publication.

While we are prepared to admit the usefulness of the *Index Medicus* to the physician engaged in literary pursuits, we cannot understand how it could reasonably be expected that any considerable number of physicians outside of the group named would pay $5 a year for the magazine in question. On the other hand, we are unable to understand why the Carnegie Institution should not continue the publication of the *Index,* paying the deficit out of its own funds. This, seems to us, is the function and purpose of that institution.

THE *Journal of Ophthalmology and Oto-Laryngology* sent out its first number last April. It is a well printed, illustrated magazine devoted to the specialties which its title indicates. We quote from its announcement the following paragraphs through which it modestly introduces itself to the profession.

The *Journal* is a monthly publication of about forty pages of original contributions, society reports, abstracts of current literature, personal mention, book reviews, etc.

The office of publication is 100 State street, Chicago, to which all communications should be addressed.

The subscription price of the *Journal* is $2 per year, sent postage free in the United States, Canada, Mexico, Cuba, and our insular possessions.

Original papers bearing on the subject of ophthalmology, otology, rhinology or laryngology are solicited with the understanding that they are contributed exclusively to this journal. Items of personal interest to the profession will be gratefully received.

---

THE second number of *Folio Therapeutica,* of which we published an advance notice in the April issue of this magazine, has come to our table. It is a periodical relating to modern therapeutici and pharmacology designed especially for medical practitioners. It bears the date of April, 1907, and opens with a letter from Professor G. A. Ewald, of Berlin, in which he states that it will be his great pleasure, from time to time, to report to the *Folia Therapeutica* new matter connected with the subject of therapeutics. Then follows an interesting group of original articles, some of which are illustrated. It contains also abstracts from articles, reviews of books, and an index of current literature on therapeutics. This magazine promises through the appearance of its initial numbers to become a valuable addition to the periodic literature of medicine.

---

THE *Detroit Medical Journal* has purchased *Leonard's Illustrated Medical Scientific Journal.* Three medical journals in Detroit have retired from the field in the last three months which is undoubtedly a good thing for Detroit and a vastly better one for the profession in general. The *Detroit Medical Journal* is to be congratulated on its acquisition. It is one of the better medical magazines of the day.

---

THE *Annals of Surgery* for June, 1907, will be a special number containing a collection of the choicest literature on modern surgery. Each article will be a practical, comprehensive treatise by an eminent specialist who has actually performed the operations described. No expense will be spared to make this the best issue, completing the forty-fifth volume.

The colored illustrations, of which there will be an abundance, have been placed in the hands of the leading medical artists of the country, and will be reproduced to the minutest detail.

It is expected that this number will at least equal that of December, 1904, which was the anniversary number of the *Annals* and which created a stir in the surgical world. The number in question will excel in point of illustraton and the price of a single copy is fixed at one dollar.

---

The *British Gynecological Journal* announces, through its editor, Dr. J. J. Macan, that it will cease publication in its present form in August next. The Obstetrical Society of London and the British Gynecological Socety will unite to form a single Section of Obstetrics and Gynecology in the Royal Society of Medicine, and the *Obstetrical Transactions* and the *British Gynecological Journal* will be superseded by the publication of the Proceedings of that Section in a form which will represent and continue both the former publications.

---

Dr. S. A. Knopf of New York, has obtained distinction in his studies of tuberculosis. He is the author of a monograph entitled "Tuberculosis as a Disease of the Masses and how to Combat it." This is an international prize essay and is now in its fourth edition, having been awarded a prize, July 31, 1900, by the International Congress on Tuberculosis. It is a monograph full of instruction for the masses, but is also interesting to physicians. It is through the wide distribution of such literature that hope may be entertained of instructing the people sufficiently to, at least, limit the awful destructiveness of pulmonary tuberculosis.

